# 

**Published:** 1887-08

**Authors:** 


CONTENTS

VOL. 34 No. 8.

| | PAGE |
|---|---|
| Mind Reading | 170 |
| Seeing by the Interior Sense | 173 |
| Healing by Laying on of Hands | 176 |
| The Comparative Energy of Nutrients | 179 |
| A Psycographic Experiment | 181 |
| Equilibrium, the Controlling Force in Nature | 182 |
| Milk and Its Products | 184 |
| Spurious Wines | 186 |
| Little Busy Bee | 188 |
| Book Notices | 188 |
| Household | 189 |
| Miscellaneous | 191 |

INDEX TO ADVERTISEMENTS OF HALF A PAGE AND OVER.

| | PAGE. |
|---|---|
| Winchester's Hypophosphate | I |
| Hollow Suppositories | II |
| Celerina | III |
| Lactopeptine | IV |
| Castoria | IV |
| Horsford's Acid Phosphate | V |
| Crystalline Phosphate | VI |
| Bovinine | VII |
| Goodyear Rubber Co. | VII |
| Lactated Food | 2d page cover |
| " " | 3d page cover |
| New York College of Magnetics | 4th page Cover |

